# Brain-spine interface: an exploration of a potential future treatment for spinal cord injury

**DOI:** 10.1007/s10143-026-04456-x

**Published:** 2026-08-22

**Authors:** Vijay Sivan, Zahin Alam, Hanish Polavarapu, Shreyes Manivel, Rohit Prem Kumar, Geoffrey R. O’Malley, Francis Ruzicka, Nitesh V. Patel

**Affiliations:** 1 Department of Neurosurgery, SUNY Upstate Medical University, Syracuse, NY USA; 2 Department of Neurosurgery, Hackensack Meridian School of Medicine, 123 Metro Blvd, Nutley, NJ 07110 USA; 3 Department of Neurosurgery, HMH-Jersey Shore University Medical Center, Neptune, NJ USA

**Keywords:** Brain-spine interface, Spinal cord injury, Neurological rehabilitation, Neurosurgery

## Abstract

Spinal cord injuries (SCIs) profoundly impact millions globally, leading to loss of motor and sensory functions below the injury site. Brain-spine interfaces (BSIs) represent an early-stage neuroprosthetic strategy that attempts to restore functional communication between cortical motor-intention signals and spinal sensorimotor circuits below the level of injury. Although early preclinical and highly selected clinical studies have shown encouraging motor outcomes, the evidence remains preliminary, and routine clinical use is limited by questions regarding safety, durability, patient selection, accessibility, and long-term functional benefit. BSI approaches are based on the observation that residual spinal pathways and sensorimotor circuits may remain partially responsive to neuromodulation even after injury. Along the way, technological advancements have significantly bolstered SCI treatment strategies, ranging from surgical interventions to regenerative therapies. Approaches such as neurostimulation and biomaterial-based strategies have shown potential in experimental and early translational settings, although their clinical efficacy and generalizability remain incompletely established. Furthermore, exploring neuroplasticity and the body’s intrinsic ability to reorganize neural connections post-injury underscores the potential for spontaneous recovery in certain cases. However, integrating BSIs into clinical practice faces substantial hurdles, including technical challenges, ethical considerations, and the need for specialized training for healthcare providers. Despite these obstacles, BSIs and other novel treatments may have potential to improve the quality of life for SCI patients, although further clinical investigation is needed to establish their safety, efficacy, and generalizability. This review catalogs recent conceptual and technological developments contributing to the emergence of BSI.

## Introduction

Paralysis affects nearly 5.4 million people in the United States, with spinal cord injury representing one of the leading causes and accounting for approximately 27.3% of cases [1, 2]. Spinal cord injuries (SCIs) result from primary mechanical disruption of axons, neurons, glia, vasculature, and spinal cord parenchyma, followed by secondary injury processes that further contribute to neurologic dysfunction. The presence of residual neural circuitry indicates that the spinal cord can still process complex sensory information despite injury, allowing for flexible treatment [3]. 

Outcomes following a spinal cord injury depend on numerous factors. The nature of the spinal cord injury plays a profound role in dictating severity, prognosis, and potential for recovery. Injuries closer to the brain, particularly cervical injuries, often yield more extensive and long-lasting impairments. This is due to proximity to critical neural pathways responsible for sensory and motor function, and autonomic control [4]. 

SCIs can be further categorized as complete or incomplete. In a complete injury, there is a total loss of sensory and motor function below the level of injury [5]. In contrast, an incomplete injury retains some degree of sensory or motor function, offering varying degrees of functional recovery. Incomplete injuries often present opportunities for neural plasticity and recovery that may not be as attainable in complete injuries.

This review focuses on current and emerging strategies for spinal cord injury management, with particular emphasis on recent advances in brain–spine interface (BSI) technologies and their potential role in restoring neurologic function.

### Literature search strategy

This narrative review was developed through a targeted search of PubMed/MEDLINE, Google Scholar, and relevant reference lists. Search terms included combinations of “spinal cord injury,” “brain-spine interface,” “brain-computer interface,” “brain-machine interface,” “epidural electrical stimulation,” “transcutaneous spinal cord stimulation,” “neuromodulation,” “functional electrical stimulation,” “neuroplasticity,” and “spinal cord rehabilitation.” The search focused primarily on English-language peer-reviewed articles published from 2000 to 2025, with earlier landmark studies included when relevant. Articles were selected based on relevance to SCI pathophysiology, current and emerging treatments, brain-spine interface mechanisms, clinical translation, and neurorehabilitation. As this was a narrative review, formal risk-of-bias assessment and meta-analysis were not performed.

## SCI biology and pathobiology

SCI is difficult to treat because the injured spinal cord develops several interacting lesion compartments, including the non-neural lesion core, the astrocytic scar border, and the surrounding reactive neural tissue. Each region contributes differently to repair failure and recovery potential [6]. The non-neural lesion core forms at the center of injury and contains inflammatory cells, fibroblast-like cells, pericytes, newly formed blood vessels, extracellular matrix, and connective-tissue-like scar components. This environment is generally poorly permissive to axonal regrowth. The astrocytic scar border forms around the lesion core and separates the damaged tissue from surrounding spared neural tissue. This border can inhibit axonal extension through extracellular matrix molecules and scar-associated inhibitors, but it also helps contain inflammation and limit lesion expansion [7]. Animal studies suggest that spared neural tissue surrounding incomplete lesions may retain greater capacity for axonal sprouting, synaptic remodeling, and circuit reorganization than tissue surrounding complete lesions [8]. 

The observation that some individuals experience partial spontaneous recovery after SCI has prompted research into the mechanisms underlying residual circuit function, neuroplasticity, and endogenous repair. Recovery depends on injury severity, neurologic completeness, injury level, age, medical comorbidities, preserved descending pathways, and the extent of spared spinal circuitry [5, 9]. Studies of neurotrophic factors, cytokine signaling, immune-cell activation, and cellular remodeling within the injured spinal cord have helped identify therapeutic targets for enhancing plasticity and limiting secondary injury [10]. Spontaneous recovery is also shaped by the temporal evolution of inflammation. In the acute phase, resident microglia activate and peripheral immune cells, including neutrophils and monocyte-derived macrophages, infiltrate the injured cord. These cells can worsen secondary injury through pro-inflammatory cytokines, reactive oxygen species, proteases, and other cytotoxic mediators [11]. During later subacute and chronic phases, immune cells may also contribute to debris clearance, extracellular matrix remodeling, and trophic support. However, persistent inflammation can maintain an inhibitory microenvironment that limits axonal regeneration and functional recovery.

Neuroregeneration after SCI is challenging because the injured spinal cord contains both intrinsic limitations to axonal growth and extrinsic barriers created by inflammation, scar formation, demyelination, and inhibitory extracellular matrix molecules. SCI pathology is commonly divided into primary and secondary injury mechanisms. Primary injury refers to the initial mechanical trauma, which disrupts axons, neurons, glia, blood vessels, and spinal cord parenchyma. Secondary injury evolves over hours to weeks and includes ionic imbalance, calcium influx, glutamate-mediated excitotoxicity, mitochondrial dysfunction, oxidative stress, blood-spinal cord barrier disruption, edema, ischemia, demyelination, apoptosis, and inflammatory-cell recruitment. Over time, astrocytes, immune cells, fibroblast-like cells, pericytes, and extracellular matrix components contribute to formation of a structured lesion border often referred to as the glial or astrocytic scar [12]. This scar has a dual role. It can inhibit axonal regrowth through physical obstruction and growth-inhibitory molecules such as chondroitin sulfate proteoglycans, but it also helps contain inflammation, restrict lesion expansion, and protect surrounding spared tissue. Neurotrophic factors such as nerve growth factor, brain-derived neurotrophic factor, and neurotrophin-3 have been investigated for their ability to support neuronal survival, axonal growth, and activity-dependent plasticity, although their effects depend on timing, delivery method, and injury model [13–15]. Overall, the immune response after SCI is dynamic rather than uniformly harmful or beneficial. Early inflammation can amplify secondary injury through cytotoxic mediators, while later immune activity may support debris clearance, tissue remodeling, and trophic signaling. However, if inflammation persists, it can sustain an inhibitory lesion environment and limit axonal regeneration. Understanding the timing, cellular composition, and molecular signaling of this immune response is essential for developing therapies that reduce secondary injury while preserving repair-supportive functions.

Contemporary translational research in SCI increasingly focuses on strategies that enhance neuroplasticity, axonal sprouting, circuit reorganization, and regeneration rather than simply preventing secondary injury. These approaches include activity-based rehabilitation, neuromodulation, molecular pathway modulation, biomaterial scaffolds, and cell-based grafting strategies. However, the translational landscape remains complex because many interventions that demonstrate axonal growth or functional recovery in animal models have not yet shown reproducible, generalizable benefit in large human clinical trials [16]. 

### Terminology and conceptual distinctions

Several related but distinct technologies are discussed in the context of SCI rehabilitation. Brain-computer interfaces (BCIs), also referred to as brain-machine interfaces (BMIs), decode neural activity to control an external device, computer, prosthesis, or stimulation system. In contrast, brain-spine interfaces (BSIs) specifically establish a closed-loop connection between decoded cortical motor intent and targeted spinal cord stimulation, thereby attempting to restore communication between supraspinal motor centers and spinal sensorimotor circuits [17]. 

Neuromodulation is a broader umbrella term that refers to therapeutic alteration of nervous system activity through electrical, magnetic, pharmacologic, or other targeted interventions [18]. Epidural electrical stimulation (EES) involves surgically implanted electrodes placed in the epidural space to stimulate spinal circuits, whereas transcutaneous spinal cord stimulation (tSCS) is a noninvasive approach that delivers stimulation through surface electrodes placed over the spine [19]. 

Functional electrical stimulation (FES) applies electrical currents to peripheral nerves or muscles to generate task-specific movements such as grasping, cycling, or stepping. Neuromuscular electrical stimulation (NMES) similarly activates peripheral nerves or muscles, often with the goal of strengthening, preventing atrophy, or improving conditioning, although it may overlap with FES when used for functional movement tasks [20]. As peripheral muscle and nerve stimulation approaches are mechanistically distinct from spinal-circuit neuromodulation and BSI, they are discussed here only to clarify terminology rather than as a major focus of the review.

## Current treatments for SCIs

The contemporary era of neurosurgery has revolutionized SCI treatment through the introduction of precise surgical techniques. Decompression and stabilization are established components of human SCI management when clinically indicated, particularly for relieving compression and restoring spinal stability. Animal studies have further helped clarify timing, mechanisms, and biological effects of decompression after SCI [21]. A limited human case series has reported functional improvements after peripheral nerve grafting combined with a biomaterial scaffold, but these findings should be interpreted cautiously because they are not yet supported by large controlled clinical trials [22, 23]. 

Similar technological leaps further amplify surgical advancements. In animal models, EES and DBS have provided mechanistic evidence that neuromodulation can influence spinal and supraspinal motor circuits after SCI. However, these findings should be distinguished from human clinical studies of EES, which have demonstrated selected functional gains in small cohorts of individuals with SCI [24–26]. Additionally, regenerative therapies, including neural progenitor cell-based approaches, have shown potential in preclinical and early translational studies, but their clinical efficacy, durability, and generalizability remain uncertain [27]. 

EES directs a current of electricity to the dorsal aspect of the spinal cord through surgically implanted electrodes [28]. Depending on the stimulation parameters, particularly frequency, there are differential patterns of movement. EES works by depolarizing large diameter afferents, which in turn activate lumbar interneurons that are involved in lower limb motor control. Mechanistic and computational studies suggest that EES activates large-diameter afferents and spinal interneuronal networks involved in lower-limb motor control. In selected human studies, EES combined with intensive rehabilitation has enabled some participants with SCI to stand, support weight, or take assisted steps [29, 30]. 

Transcranial magnetic stimulation (TMS), particularly high-frequency repetitive TMS (HF-rTMS), has emerged as a noninvasive approach with potential to modulate corticospinal excitability and promote activity-dependent plasticity. In experimental models, activation of MAP2K signaling through genetic engineering or HF-rTMS promoted corticospinal axon sprouting and functional regeneration, suggesting a potential translational pathway for enhancing endogenous repair mechanisms after SCI. However, these findings remain primarily experimental and require further validation in human SCI populations [31]. 

Robotic therapy for SCIs relies on a device that stimulates repetitive proprioceptive input from the limbs [32]. This input may promote neuroplasticity, defined here as activity-dependent reorganization and strengthening of spared neural pathways through repeated sensory feedback, motor practice, and reinforcement of residual spinal and supraspinal circuits. These activity-dependent interventions aim to strengthen residual motor pathways and improve functional limb movement. In human rehabilitation studies and meta-analyses, robot-assisted gait training has been associated with improvements in walking ability and lower-limb strength in some individuals with SCI, although outcomes vary by injury completeness, baseline function, and training intensity [33]. 

There are a number of pharmacological interventions designed to address SCIs. Some examples include minocycline, fampridine, and hepatocyte growth factor (HGF) [34, 35]. Minocycline targets multiple processes that are involved in mediating cell death and prevents the progression of secondary injury following a spinal cord injury [34]. Some pharmacologic agents have been evaluated in human or clinical contexts, including minocycline and fampridine, although efficacy remains variable and not universally established. Other agents, such as HGF, have shown regenerative potential in experimental and early translational settings, but they should not be presented as established clinical therapies for SCI [35]. 

Cell-based approaches, such as human embryonic stem cells, adult stem cells, and fetal-derived neural cells remain an important area of SCI research, particularly for axonal regeneration, remyelination, and modulation of the post-injury microenvironment [36–38]. Similarly, biomaterial scaffolds and hydrogels may support SCI repair by bridging lesion cavities, modulating scar formation, or delivering cells, growth factors, or drugs [39]. 

NMES is used to increase the strength of partially paralyzed muscles in people who are affected by SCIs [40]. Combining NMES with FES has been shown to induce hypertrophy in weakened muscles [40]. tSCS is a noninvasive neuromodulatory technique that delivers electrical stimulation to spinal sensorimotor circuits through surface electrodes placed over the skin, typically overlying the targeted spinal segments. When combined with intensive rehabilitation, tSCS may enhance motor function by promoting activity-dependent neuroplasticity [41]. Combining this procedure with intense exercise aims to restore movement and function through neuroplasticity [42]. 

Together, these treatment strategies demonstrate the multifaceted progress being made in SCI management while also highlighting the ongoing need for approaches that can more directly integrate neural signal decoding, targeted stimulation, and activity-dependent rehabilitation, as seen in emerging closed-loop neuromodulation and BSI technologies.

## Emerging technologies

To avoid conflating related but distinct approaches, emerging technologies for SCI can be organized into four broad categories: invasive brain-spine interfaces, non-invasive brain-spine interfaces, spinal neuromodulation without cortical decoding, and other related neuromodulatory strategies that may influence locomotor recovery but do not themselves constitute BSIs. This distinction is important because true BSIs require both neural signal decoding and stimulation of spinal circuits, whereas many neuromodulation approaches influence spinal or supraspinal circuits without creating a real-time brain-to-spine bridge.

### Conceptual framework of brain-spine interfaces

The development of BSIs and related neuromodulation devices represents an emerging area of investigation in SCI treatment. Whereas conventional BCIs or BMIs often decode neural signals to control external devices such as computers, cursors, or prostheses, BSIs are designed to restore functional communication between supraspinal motor centers and spinal sensorimotor circuits below the level of injury. In experimental and early clinical settings, BSIs are designed to translate neural activity related to intended movement into stimulation commands that modulate spinal circuits in real time [43]. The function of these machines is refined by artificial intelligence and machine learning, allowing for personalized rehabilitation regimens [44–46]. 

**Fig. 1:**
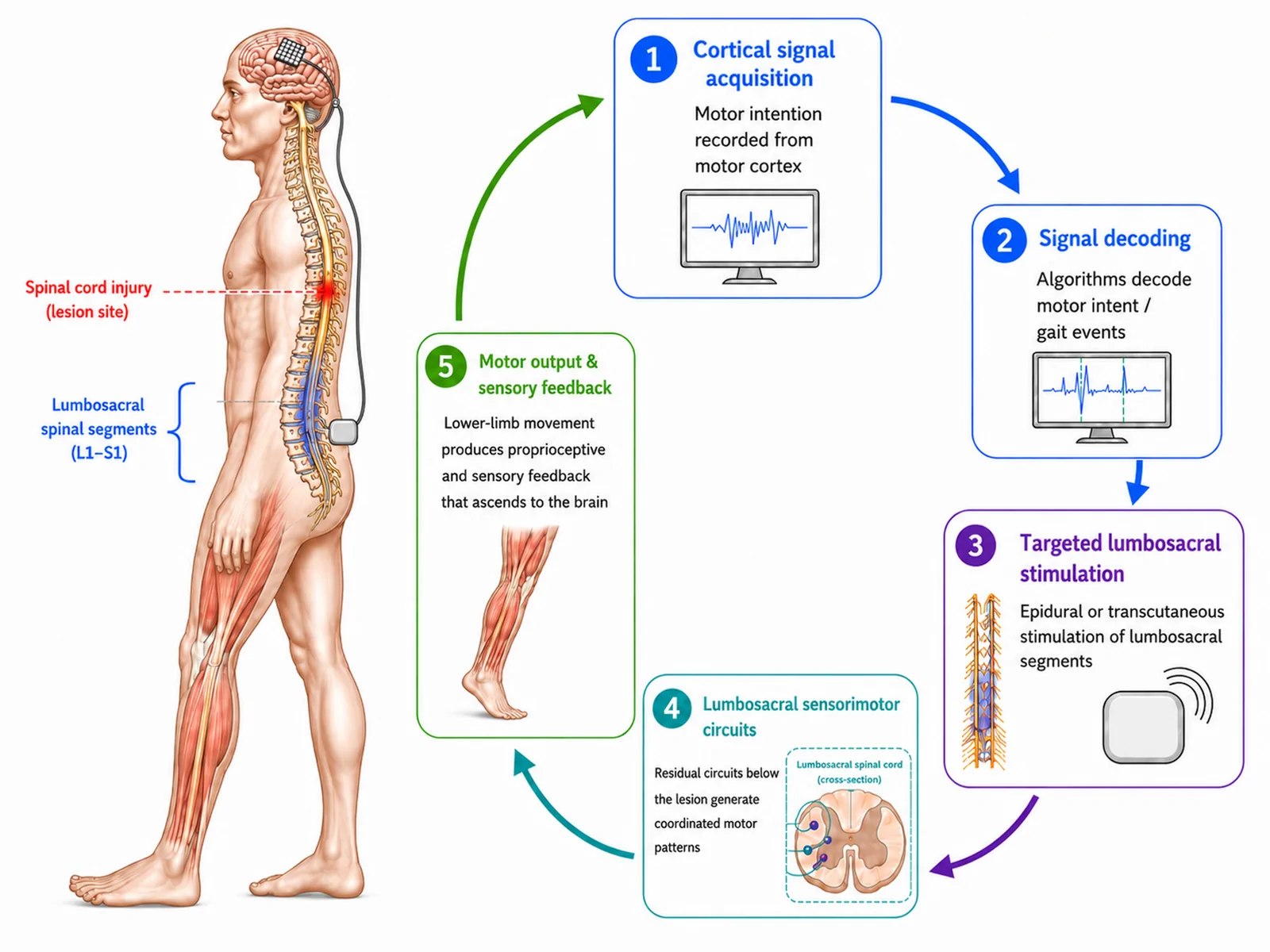
**General closed-loop brain–spine interface framework for lower-limb restoration after SCI. **The schematic represents a general BSI framework and demonstrates cortical motor-intention signal acquisition, real-time decoding into motor commands or gait events, targeted epidural or transcutaneous stimulation of the lumbosacral spinal segments, activation of residual lumbosacral sensorimotor circuits, and lower-limb motor output with ascending sensory feedback

BSI specifically aims to establish a functional link between supraspinal motor centers and spinal sensorimotor circuits below the level of injury. Mechanistically, a BSI should be understood as a brain-to-spine control loop. First, cortical activity associated with intended movement is acquired from the motor cortex, either through implanted cortical electrodes in invasive systems or scalp-based recordings such as EEG in non-invasive systems. Second, decoding algorithms identify patterns of neural activity and translate them into predicted motor commands, gait-phase events, or limb trajectories, such as hip flexion, knee extension, foot strike, or foot-off. Third, the decoded output is converted into stimulation commands that drive targeted spinal stimulation, commonly through EES or tSCS [47, 48]. Fourth, spinal stimulation engages residual lumbosacral sensorimotor circuits, including dorsal-root afferents, interneuronal networks, and motor pools below the lesion. Fifth, activation of these circuits produces task-specific motor output when paired with attempted movement. Moreover, the resulting movement also produces proprioceptive, cutaneous, visual, and biomechanical feedback that may contribute to closed-loop adjustment and activity-dependent plasticity during rehabilitation (Fig. 1) [48–50]. 

### Invasive brain-spine interfaces

Invasive BSIs represent the most direct form of brain-spine interfacing because they combine implanted cortical recording systems with targeted spinal stimulation to restore communication between motor intent and spinal sensorimotor circuits. The preclinical and early clinical literature should be separated carefully because different studies use different signal sources, decoding approaches, stimulation targets, and outcome measures.

Capogrosso et al. demonstrated a wireless BSI in a nonhuman primate model in which intracortical recordings from the motor cortex were decoded in real time to identify gait events and control epidural stimulation of lumbar spinal circuits. This work showed that cortical activity could be translated into stimulation commands that restored weight-bearing locomotor patterns in an experimental paralysis model. However, its limitations include its preclinical design, controlled experimental setting, and uncertain generalizability to chronic human SCI [50]. 

Bonizzato et al. further supported the mechanistic importance of closed-loop timing by showing that brain-controlled modulation of spinal circuits may enhance locomotor recovery compared with non-contingent or continuous stimulation in an animal model. This finding reinforces a key BSI principle: stimulation is most biologically meaningful when it is linked to neural intent or task phase rather than delivered as isolated background neuromodulation. However, these data remain preclinical and require confirmation in larger human studies [51]. 

Lorach et al. later translated this concept into a first-in-human/single-participant BSI system in a patient with chronic SCI. In that study, cortical activity related to intended lower-limb movement was recorded wirelessly and decoded into stimulation commands delivered to lumbosacral epidural electrodes. The system enabled more natural walking with crutch support and improved performance on tasks such as ramp walking and stair climbing. When the BSI was inactive, walking ability was lost despite cortical activity indicating gait initiation, supporting the importance of the decoded brain-to-spine bridge. The participant also demonstrated improvements in sensory, motor, and clinical assessments after BSI-mediated rehabilitation, suggesting possible additive neurological recovery [52]. 

Despite these encouraging findings, this evidence remains highly selected and should not be interpreted as broadly generalizable clinical efficacy. Key limitations include the single-participant design, need for multiple implanted devices, intensive calibration and rehabilitation, long-term durability concerns, and uncertain applicability across different injury levels, chronicity, residual descending pathways, and completeness of SCI. Larger prospective studies are needed before BSIs can be considered ready for routine clinical use.

### Non-invasive brain-spine interfaces

Non-invasive BSI approaches seek to achieve a similar brain-to-spine connection without implanted cortical electrodes, most commonly by pairing electroencephalography-based decoding of motor intent with tSCS. In 2025, Atkinson et al. introduced and evaluated a novel non-invasive BSI that integrates electroencephalography decoding of motor intent with tSCS to facilitate movement. In this proof-of-concept study, six able-bodied participants performed voluntary knee extension tasks while the system detected µ- and β-band event-related desynchronization from the sensorimotor cortex to trigger tSCS in real time. The BSI achieved robust classification accuracy, with an average area under the curve of approximately 0.83 during cued tasks and 0.68 during uncued tasks, and was well tolerated without adverse effects. By demonstrating the feasibility of coupling cortical signals with spinal stimulation without surgical intervention, this work expands the potential for accessible neurorehabilitation strategies aimed at restoring voluntary motor function. However, this study primarily focuses on able-bodied participants rather than individuals with SCI, and thus these results demonstrate more proof-of-concept than direct clinical evidence [53]. 

Additionally, a recent translational proof-of-concept study used non-invasive human intention-related signals, including EOG/EEG-based control signals, to drive epidural spinal stimulation in an anesthetized macaque, producing left- and right-sided stepping-like lower-limb movements. The system used a portable non-invasive sensor and achieved an average decoding F1 score of 89.6% across four control commands. However, because the locomotor output was generated in an anesthetized macaque rather than a human SCI participant, this study should be interpreted as translational proof-of-concept [54]. 

### Comparative evidence across brain-spine interface approaches

Overall, the current BSI literature can be organized by signal source, decoding strategy, stimulation modality, feedback loop, study population, functional outcome, and limitation. Invasive systems provide higher-resolution cortical signals and more spatially targeted epidural stimulation, but they require neurosurgical implantation and raise concerns regarding infection, hardware longevity, signal stability, and cost. Non-invasive systems reduce surgical burden by using EEG-based motor-intention decoding and transcutaneous stimulation, but they generally face lower signal resolution, reduced stimulation specificity, and greater susceptibility to artifact. Preclinical systems provide important mechanistic proof-of-concept, whereas human evidence remains limited to able-bodied feasibility studies, translational human-animal demonstrations, and highly selected early clinical cases. Therefore, the key translational question is not only whether BSI can generate movement under controlled conditions, but whether it can produce durable, reproducible, patient-centered functional gains across heterogeneous SCI populations.

A study-level comparison clarifies these distinctions. Capogrosso et al. used invasive cortical recordings in nonhuman primates to decode gait events and control lumbar epidural stimulation, demonstrating restoration of locomotor patterns in a preclinical model. Bonizzato et al. showed that brain-controlled modulation of spinal circuits could improve recovery compared with non-contingent stimulation in an animal model, supporting the importance of timing stimulation to neural intent. Lorach et al. provided first-in-human evidence that decoded cortical intent can drive lumbosacral epidural stimulation to support walking after SCI, but the report remains limited by its single-participant design. Atkinson et al. demonstrated the feasibility of a non-invasive EEG-tSCS interface in able-bodied participants, showing that motor-intention signals can trigger spinal stimulation without implanted cortical electrodes, although direct evidence in SCI patients remains limited. Mo et al. further supported proof-of-concept feasibility by using non-invasive human intention-related EOG/EEG signals decoded in real time to control epidural spinal stimulation in an anesthetized macaque, producing left- and right-sided stepping-like lower-limb movements. However, because the motor output occurred in an anesthetized nonhuman primate rather than in a human participant with SCI, the findings should be interpreted as translational proof-of-concept rather than direct clinical evidence (Table 1) [50–54]. 

**Table 1:** **Study-level comparison of brain-spine interface approaches. **Major BSI-related studies are organized by evidence level, signal source, cortical recording site, stimulation target, population or injury model, functional outcome, and key limitations to clarify the translational status of current BSI research

Study type	Signal source / electrode	Cortical site	Stimulation target	Population/model	Main outcome	Limitation
Capogrosso et al.	Intracortical recordings	Motor cortex	Lumbar epidural stimulation	Nonhuman primates	Improved locomotor patterns	Preclinical model
Lorach et al.	Implanted cortical recording system	Motor cortex	Lumbosacral epidural stimulation	Single human participant with chronic SCI	Walking with crutch support; ramp/stair tasks	Single participant; highly selected
Atkinson et al.	EEG	Sensorimotor cortex	tSCS	Able-bodied participants	Feasibility of non-invasive BSI triggering	Not studied in SCI patients
Mo et al.	EOG/EEG	Non-invasive intention-related signals	Epidural stimulation	Human intention-related signals controlling stimulation in an anesthetized macaque	Stepping-like movements in anesthetized macaque	Translational proof-of-concept; not direct human SCI evidence

### Spinal neuromodulation without cortical decoding

Spinal neuromodulation without cortical decoding represents a related but distinct strategy in which epidural or transcutaneous stimulation is used to modulate spinal circuits without real-time input from decoded brain activity [55]. These approaches helped establish the therapeutic potential of spinal circuit activation after SCI and provide an important foundation for BSI systems, but they do not themselves constitute BSIs unless stimulation is driven by decoded neural intent.

### Related neuromodulation strategies

In 2024, Cho et al. studied lateral hypothalamic deep brain stimulation (DBSLH) in two individuals with incomplete spinal cord injury who had persistent gait deficits despite standard rehabilitation. Although not a BSI, lateral hypothalamic deep brain stimulation represents a related neuromodulatory strategy that may influence locomotor recovery after SCI. DBSLH produced immediate improvements in lower limb muscle activity, kinematics, endurance, and reduced perceived walking effort, and after three months of combined DBS and gait training, participants showed better walking performance and motor scores. Notably, these long-term gains persisted even when the DBS was turned off and were achieved without adverse effects on vital signs, weight, or hormones [56] (Table 2). 

**Table 2:** Key milestones in spinal neuromodulation and brain-spine interface technologies for SCI

Approach	Role in SCI/BSI development	Evidence status	Key translational barriers
Epidural electrical stimulation without cortical decoding	Demonstrated that spinal sensorimotor circuits below the lesion can be activated to support standing, stepping, and voluntary movement in selected patients.	Small human studies, case reports, and mechanistic studies; not broadly generalizable.	Surgical invasiveness, patient selection, stimulation optimization, rehabilitation intensity, durability, complications, and cost.
Transcutaneous spinal cord stimulation	Established a noninvasive method for modulating spinal excitability through surface electrodes over targeted spinal segments.	Early clinical and rehabilitation studies with heterogeneous protocols and outcomes.	Variable targeting, durability of benefit, protocol standardization, patient selection, and need for intensive rehabilitation.
BCI/BMI cortical decoding	Enabled decoding of motor intent from cortical activity to control external devices such as computers, cursors, robotic limbs, or prostheses.	Established proof-of-concept and clinical research base, but often focused on external device control rather than spinal circuit restoration.	Signal stability, decoding accuracy, implant burden for invasive systems, training demands, and long-term usability.
Invasive brain-spine interfaces	Combine cortical signal acquisition, real-time decoding, and targeted spinal stimulation to reconnect motor intent with spinal sensorimotor circuits.	Strongest BSI-specific evidence, but still limited to preclinical studies and early first-in-human/single-participant reports.	Implant burden, decoding stability, long-term safety, infection risk, hardware durability, rehabilitation demands, cost, and regulation.
Non-invasive brain-spine interfaces	Pair EEG-based motor-intention decoding with noninvasive spinal stimulation, such as tSCS, to reduce surgical burden.	Proof-of-concept evidence, including able-bodied participant studies; limited direct evidence in SCI patients.	Lower signal resolution, decoding reliability, stimulation specificity, reproducibility, and need to demonstrate clinical benefit in SCI.
Spinal neuromodulation without cortical decoding	Shows that spinal circuits can be therapeutically activated without real-time brain-derived input, providing a foundation for BSI development.	Human and preclinical evidence for EES/tSCS; not a BSI unless stimulation is driven by decoded neural intent.	Lack of cortical intent integration, protocol heterogeneity, uncertain long-term outcomes, and individualized stimulation needs.
Related supraspinal neuromodulation, including lateral hypothalamic DBS	Suggests that non-BSI brain stimulation approaches may influence locomotor recovery and broader rehabilitation pathways after SCI.	Very early evidence from small cohorts; conceptually distinct from BSI.	Small sample sizes, unclear mechanisms, invasiveness, generalizability, patient selection, and need for larger controlled studies.
Clinical translation of BSI systems	Represents the movement from experimental closed-loop systems toward clinically deployable neurorehabilitation tools.	Not ready for routine clinical use; current evidence remains proof-of-concept or highly selected.	Patient selection, injury heterogeneity, chronicity, residual circuitry, long-term follow-up, ethics, privacy, cybersecurity, reimbursement, infrastructure, and regulation.

## Future directions

Despite promising advances, integrating BSIs into standard clinical practice is fraught with technical, ethical, and logistical challenges. Importantly, current clinical evidence should be interpreted cautiously, as much of the existing literature remains proof-of-concept, preclinical, or based on single-patient reports and small cohorts. Although early studies have demonstrated encouraging improvements in voluntary movement, gait, and patient autonomy, these findings may not be broadly generalizable across the heterogeneous SCI population. Therefore, therapeutic claims regarding BSI, EES, DBS, regenerative therapies, and other emerging interventions should be interpreted according to the level of evidence supporting each approach, which ranges from preclinical proof-of-concept to small human cohorts and highly selected early clinical reports. Clinical translation will also require clearer patient selection criteria, particularly regarding injury level, completeness of injury, time since injury, preserved descending or propriospinal pathways, medical comorbidities, and ability to participate in intensive rehabilitation. The invasiveness of implanted cortical and spinal devices must also be weighed against potential functional benefit. Moreover, differences in injury level, completeness of injury, chronicity, residual neural circuitry, rehabilitation intensity, and device configuration may substantially influence outcomes. Additionally, the precision in recording and interpreting neural signals, the durability and safety of implantable devices, and the efficacy of non-invasive methods pose significant hurdles [57, 58]. Additionally, the implications of neural manipulation, patient consent, and the long-term impact of BSI use require careful consideration [59]. Long-term safety, durability of functional gains, device reliability, infection risk, hardware failure, and the need for revision procedures remain incompletely characterized. Furthermore, the extent to which BSI-mediated improvements persist after stimulation is discontinued, or translate into meaningful gains in activities of daily living, requires further investigation in larger prospective studies.

Logistically, the accessibility of BSI technology, cost implications, and the training of specialized personnel present additional barriers. Regulatory considerations, including device approval pathways, cybersecurity, data privacy, long-term monitoring, adverse-event reporting, and standards for explantation or device revision, will also be essential before widespread clinical implementation. Moreover, there is a pressing need to delve deeper into the patient’s perspective. Those who have undergone implantation of BSIs in clinical trials have reported experiencing heightened autonomy and a greater sense of self-control. However, contrasting experiences have been expressed, with some individuals describing postoperative feelings of detachment and challenges in integrating the implanted devices into their sense of self [59]. 

Future research should prioritize adequately powered clinical trials with standardized outcome measures, longer follow-up periods, and careful reporting of adverse events to better define patient selection criteria, reproducibility, and real-world clinical benefit. These challenges underscore the complexity of clinically incorporating BSI, highlighting the need for continued innovation to realize this technology’s full potential in transforming individuals’ lives.

## Conclusion

Spinal cord injury treatment has progressed from supportive care and surgical stabilization toward increasingly sophisticated strategies that seek to preserve neural tissue, modulate residual circuits, promote plasticity, and restore functional movement. BSIs represent a promising engineered neuroprosthetic approach that attempts to bridge disrupted communication between cortical motor-intention signals and spinal sensorimotor circuits below the level of injury. However, BSIs should not be interpreted as a natural reconnection of the brain and spine, nor are they ready for routine clinical use. Current BSI evidence remains early-stage and is largely derived from preclinical studies, proof-of-concept systems, small cohorts, and highly selected participants. Important limitations include patient selection, invasiveness, surgical and device-related risks, long-term safety, decoding stability, durability of benefit, rehabilitation intensity, cost, accessibility, and uncertain generalizability across the broader SCI population. Future studies will need to establish whether BSI-mediated gains are reproducible, durable, clinically meaningful, and achievable outside specialized research settings. Thus, while BSIs may become an important component of future SCI rehabilitation, their promise must be balanced against the substantial technical, clinical, ethical, and logistical barriers that remain.

## Data Availability

No datasets were generated or analysed during the current study.
